# Analysis of a logical regulatory network reveals how Fe-S cluster biogenesis is controlled in the face of stress

**DOI:** 10.1093/femsml/uqad003

**Published:** 2023-03-02

**Authors:** Firas Hammami, Laurent Tichit, Béatrice Py, Frédéric Barras, Pierre Mandin, Elisabeth Remy

**Affiliations:** Laboratoire de Chimie Bactérienne (UMR7283), IMM, IM2B, CNRS, Aix-Marseille University, 13009 Marseille, France; I2M, CNRS, Aix-Marseille University, 13009 Marseille, France; I2M, CNRS, Aix-Marseille University, 13009 Marseille, France; Laboratoire de Chimie Bactérienne (UMR7283), IMM, IM2B, CNRS, Aix-Marseille University, 13009 Marseille, France; Institut Pasteur, Département de Microbiologie, Université Paris‐Cité, UMR CNRS 6047, SAMe Unit, F-75015 Paris, France; Laboratoire de Chimie Bactérienne (UMR7283), IMM, IM2B, CNRS, Aix-Marseille University, 13009 Marseille, France; I2M, CNRS, Aix-Marseille University, 13009 Marseille, France

**Keywords:** mathematical modeling, regulatory networks, bacterial regulation, logical modeling, IscR, Fe-S cluster biogenesis

## Abstract

Iron-sulfur (Fe-S) clusters are important cofactors conserved in all domains of life, yet their synthesis and stability are compromised in stressful conditions such as iron deprivation or oxidative stress. Two conserved machineries, Isc and Suf, assemble and transfer Fe-S clusters to client proteins. The model bacterium *Escherichia coli* possesses both Isc and Suf, and in this bacterium utilization of these machineries is under the control of a complex regulatory network. To better understand the dynamics behind Fe-S cluster biogenesis in *E. coli*, we here built a logical model describing its regulatory network. This model comprises three biological processes: 1) Fe-S cluster biogenesis, containing Isc and Suf, the carriers NfuA and ErpA, and the transcription factor IscR, the main regulator of Fe-S clusters homeostasis; 2) iron homeostasis, containing the free intracellular iron regulated by the iron sensing regulator Fur and the non-coding regulatory RNA RyhB involved in iron sparing; 3) oxidative stress, representing intracellular H_2_O_2_ accumulation, which activates OxyR, the regulator of catalases and peroxidases that decompose H_2_O_2_ and limit the rate of the Fenton reaction. Analysis of this comprehensive model reveals a modular structure that displays five different types of system behaviors depending on environmental conditions, and provides a better understanding on how oxidative stress and iron homeostasis combine and control Fe-S cluster biogenesis. Using the model, we were able to predict that an *iscR* mutant would present growth defects in iron starvation due to partial inability to build Fe-S clusters, and we validated this prediction experimentally.

## Author summary

Iron sulfur (Fe-S) clusters appeared early in life, when oxygen tension was low and iron plentiful, and have been used since as cofactors for a wide variety of proteins involved in a plethora of reactions. However, synthesis and stability of Fe-S clusters is compromised in conditions where iron is low or in presence of reactive oxygen species. Living organisms have developed complex regulatory networks to allow biogenesis of Fe-S clusters in function of environmental conditions. Thus, understanding this regulation is of primary importance as changes in Fe-S cluster biogenesis impact the physiology of organisms and is for instance involved in resistance of bacteria to certain antibiotics. We here used a modeling approach to gain a global systemic understanding of the process. We developed a mathematical logical model which extensively describes the regulatory network that controls biogenesis of Fe-S clusters in the model bacterium *Escherichia coli*. Analysis of the model reveals how Fe-S biogenesis is organized in function of environmental conditions and reveals how oxidative stress and iron homeostasis combine and control Fe-S cluster biogenesis.

## Teaser

Mathematical modeling of Fe-S cluster biogenesis in Escherichia coli reveals how oxidative stress and iron homeostasis combine and control Fe-S cluster biogenesis, allowing phenotypic predictions.

## Introduction

Iron sulfur (Fe-S) clusters are ubiquitous prosthetic groups formed of two to four iron centers covalently bound by sulfur atoms. Fe-S clusters containing proteins are present in virtually all living organisms and are involved in a plethora of biological processes ranging from respiration to the TCA cycle, gene regulation or bacterial virulence (Roche et al. 2013). However, Fe-S clusters can be extremely sensitive to environmental stresses, in particular iron deprivation and oxidative stress. Indeed, iron is needed to assemble Fe-S clusters, while Reactive Oxygen Species (ROS) damage solvent exposed Fe-S clusters, like in dehydratases (Imlay 2006, Jang and Imlay 2010, Baussier et al. 2020). An additional level of complexity comes from the fact that iron and ROS are linked through the Fenton reaction:


\begin{eqnarray*} {\rm Fe}^{\rm 2+} + {\rm H}_{\rm 2}{\rm O}_{\rm 2} \to {\rm Fe}^{\rm 3 +} + {\rm HO}^{\rm -} + {\rm HO}^\cdot \end{eqnarray*}

This reaction leads to the production of highly reactive hydroxyl radicals for which no direct scavenger has yet been described. Fe-S cluster biogenesis is thus tightly regulated by actors of the iron homeostasis and oxidative stress responses involving multiple regulators of different nature (transcription factors and regulatory RNAs). Altogether, this complex regulatory network makes it difficult to predict how the cell will adapt Fe-S clusters demand in face of stresses encountered in nature.

Control of Fe-S clusters biogenesis has been best studied in the model bacterium *Escherichia coli*, that can adapt to multiple niches wherein iron and oxygen concentrations can vary greatly. Here, we aimed at deciphering the regulatory network controlling Fe-S clusters biogenesis of *E. coli* through a mathematical modelling approach. Indeed, mathematical models reveal global dynamical behaviors emerging from regulatory networks. Model analysis untangles the contribution of the different regulators, identifies key actors, and allows to make predictions on how biological systems respond to environmental and/or genetic perturbations.

In *E. coli*, Fe-S clusters biogenesis is ensured by two machineries, Isc and Suf, present in a wide variety of organisms from bacteria to eucaryotes (Roche et al. 2013, Baussier et al. 2020, Garcia et al. 2022). While the Isc and Suf machineries consist of different macromolecular complexes, the strategies used for Fe-S cluster biogenesis are similar in both systems. In the first step, iron and sulfur are acquired (through a yet unknown iron donor, and from cysteine, respectively) and then assembled on a scaffold complex. The formed Fe-S cluster is then delivered to apo-proteins through A-Type carriers (ATC) (Roche et al. 2013). In addition to ATCs of the main Fe-S cluster biogenesis machineries, IscA and SufA, *E. coli* displays non-Isc non-Suf carriers such as ErpA and NfuA that serve to multiply the Fe-S cluster transport routes in function of environmental conditions (Vinella et al. 2009). In particular, *erpA* is essential in aerobiosis, and NfuA is necessary for targeting clusters under iron starvation or oxidative stress (Loiseau et al. 2007, Angelini et al. 2008, Py et al. 2018).

In *E. coli*, Fe-S cluster biogenesis is mainly regulated by the IscR transcription factor, itself an Fe-S cluster containing protein matured almost exclusively by the Isc machinery (Schwartz et al. 2001, Giel et al. 2006, Yeo et al. 2006, Mettert and Kiley 2014, Mettert and Kiley 2015). In its holo (Fe-S cluster bound) form, IscR represses the transcription of several genes among which the *iscRSUA* operon, encoding components of the Isc machinery, and the *erpA* and *nfuA* genes. In contrast, accumulation of apo-IscR form activates transcription of the *sufABCDSE* operon, encoding the Suf machinery. In this way, both the Fe-S cluster state and the concentration of IscR dictate which Fe-S cluster biosynthesis machinery and carriers are being used by *E. coli*.

Other global regulators are involved in the choice between Isc and Suf. In *E. coli*, iron homeostasis is mainly controlled by the Fur transcription factor and by the non-coding regulatory RNA RyhB (Chareyre and Mandin 2018). When bound to iron, Fur represses iron import genes and inhibits Suf and RyhB expression (Seo et al. 2014). Iron starvation alleviates Fur mediated repression of the aforementioned genes. RyhB induction causes iron sparing, both by inhibiting translation of non-essential iron using proteins and by promoting siderophores production (Prévost et al. 2007, Seo et al. 2014, Beauchene et al. 2015, Chareyre and Mandin 2018). RyhB base-pairs to the *iscRSUA* mRNA and inhibits expression of the Isc machinery while leaving that of IscR intact (Desnoyers et al. 2009). In this way RyhB was proposed to indirectly lead to the accumulation of apo-IscR and to Suf expression.

Among the several oxidative challenges that can affect Fe-S clusters, we focus on H_2_O_2_ since it can react with the iron of Fe-S clusters to fuel the Fenton reaction. Response to H_2_O_2_ driven oxidative stress is mainly regulated by OxyR in *E. coli* (Aslund et al. 1999). OxyR activation by H_2_O_2_ leads to the induction of the *suf* operon (Jang and Imlay 2010). In addition, OxyR also activates the expression of the catalase KatG and peroxidase AhpCF (together annotated Hpx), that diminish H_2_O_2_ concentration, as well as the expression of Dps and YaaA, that store the free intracellular iron in order to limit Fenton reaction (Park et al. 2005, Yeo et al. 2006, Liu et al. 2011). Moreover, OxyR induces Fur expression to limit the ROS-dependent demetallation of Fur (Varghese et al. 2007).

Mathematical modelling is increasingly used to formalize, synthesize and analyze set of regulatory interactions controlling biological processes. We present here, a qualitative mathematical model encompassing the molecular actors of Fe-S cluster biogenesis regulation, iron homeostasis, and oxidative stress response based on the logical formalism. This model explains mechanistically how the two main systems Isc and Suf are coordinated in response to external iron and oxygen signals. We then determined the individual and combined contributions of oxygen and iron on Fe-S cluster biogenesis, before predicting situations of Fe-S cluster dependent growth defects in mutants.

## Results

### Establishment of a logical model for the Fe-S cluster biogenesis

**Figure 1.**
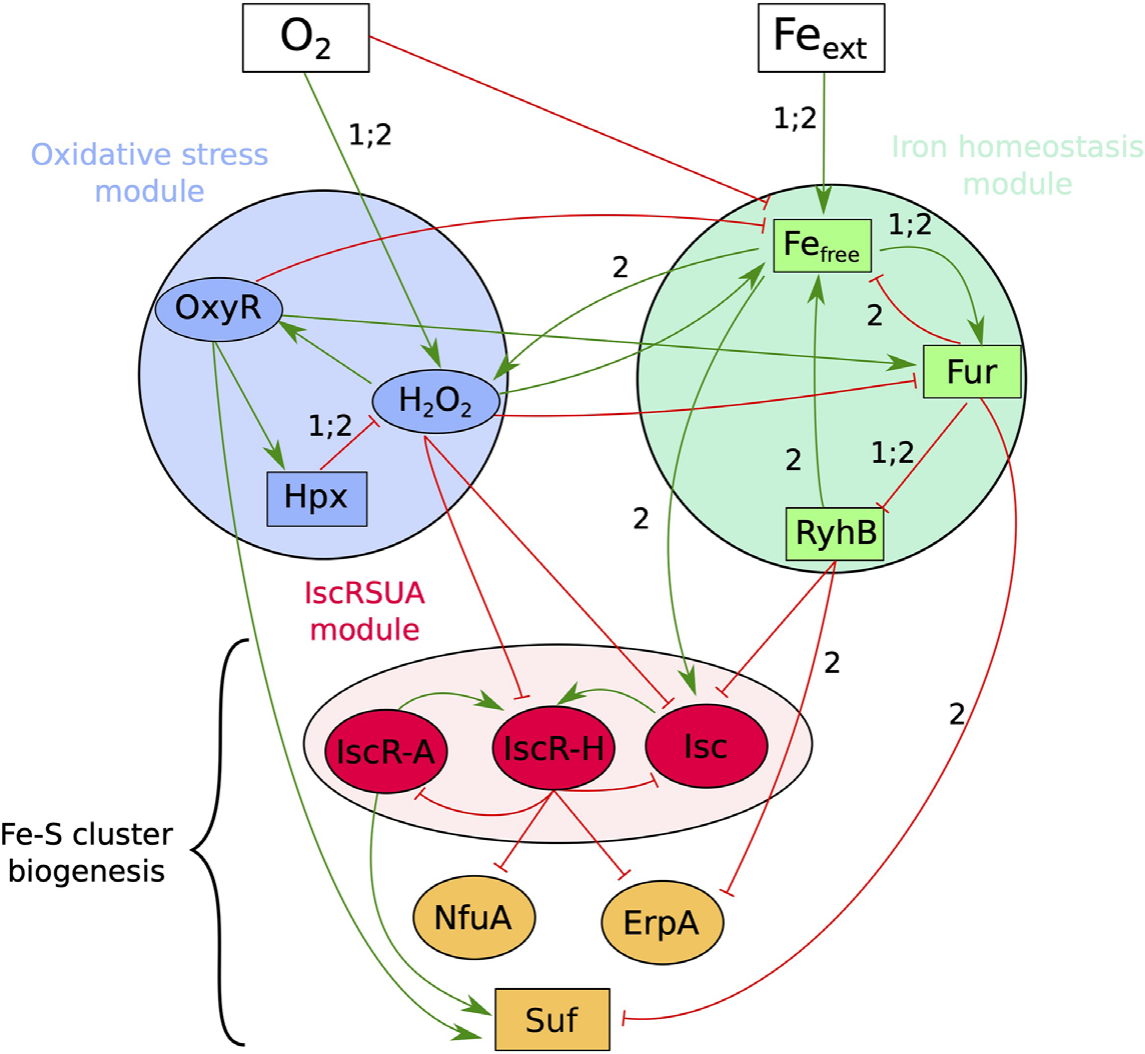
Regulatory Graph centered on the Fe-S cluster biogenesis in *E. coli*. Nodes represent biological components, and edges regulations: activations (green, normal arrow) or inhibitions (red, T-shaped arrows). Ellipses stand for Boolean nodes (i.e. value of the node can only be 0 or 1), rectangles for multivalued nodes. Labels on edges specify the regulatory thresholds (equal to 1 if not indicated); two labels on the same edge means the regulation happens at both thresholds. Each module is represented by a color: blue nodes to the oxidative stress module; green nodes to the iron homeostasis module; the red nodes represent the IscRSUA module. The three orange nodes are the outputs of the model.

We built a gene regulatory network describing Fe-S cluster biogenesis regulation in response to iron and oxygen concentrations in the environment. It consists in a directed signed graph where nodes represent genes or biological components, and edges the positives or negative influences (Materials and Methods and Table 1 for definition of key mathematical concepts used hereafter). The model encompasses the three biological processes described above: iron homeostasis, with the nodes *Fe_free_, Fur* and *RyhB;* oxidative stress with nodes *H_2_O_2_, OxyR* and *Hpx*, which represents the catalases and peroxidases involved in H_2_O_2_ detoxification; Fe-S clusters biogenesis with the nodes *IscR-A, IscR-H, Isc, Suf, NfuA* and *ErpA*. Input nodes *Fe_ext_*and *O*_2_ stand for external iron and oxygen. Note that we chose to describe the IscR regulator with two nodes, *IscR-A* (for apo IscR) and *IscR-H* (for holo-IscR) to describe both the expression and the maturation of the protein. The three output nodes *ErpA, NfuA*, and *Suf*, and also node *Isc* serve as read-out of Fe-S cluster biogenesis. The resulting model is composed of 14 nodes and 28 directed interactions (Fig. 1). A full description of the nodes of the model is provided in the Material and Methods section.

**Table 1.** Definition of key mathematical concepts used in this study

**Regulatory graph (RG)**	A graph that represents a genetic interaction network. **Nodes** of the RG represent the biological components (proteins, regulatory RNAs, molecules…), and **edges** the regulations between components. Edges are directed, and labeled to specify the level at which the regulation occurs (1 by default).
**Discrete variable**	A variable that can take only a finite number of values. In logical formalism, we attach such a discrete variable to each node in order to represent its **qualitative activity level**. A Boolean variable can take two values: 0 means the component is not active (i.e. not able to regulate its targets), 1 means the component is active.
**State Transition Graph (STG)**	A directed graph that represents the possible discrete trajectories of the system. **Nodes** of the STG represent the **states** of the system, i.e. a vector giving the values of each component, and **edges** the transitions between states.
**Asymptotic behavior**	Long-time behavior of the system when it tends to an equilibrium for a given set of input values. It allows to determine which state(s) the system reaches and which components stabilize.
**Attractor**	An attractor is a set of states which, once reached, will be visited indefinitely by the system: the dynamics is confined in this subspace and stabilizes. Two types of attractors can be distinguished:
	-**Stable states**: all the components of the state are stable, the global system does not evolve anymore;
	-**Complex attractors**: some of the components show oscillations and others are stable. These oscillations may be the result of homeostatic regulations that maintain the system around an equilibrium. .

We parameterized this graph to integrate its dynamics (see Materials and Methods). Half of the components are associated with Boolean variables (representing the inactivity/activity state of the components), and half with ternary variables representing low, medium and high activity levels, except for two nodes: the *O*_2_ node for which the 3 levels indicate anaerobic, aerobic, and oxidative stress conditions; the *Hpx* node for which the basal level is 1 (representing constitutive expression of catalases that scavenge low doses of H_2_O_2_ produced by respiration), level 2 represent high expression of catalases during oxidative stress, and level 0 is used to simulate knock-out (KO) mutation. The variables of the model are described in Table S1. Logical equations are settled on the basis of available biological knowledge (Table 2). For example, the node *IscR-H*, which represents holo-IscR, is activated by the presence of both nodes *Isc* and *IscR-A*. Indeed, *in vivo* the Isc machinery is required for the maturation of the apo form of IscR into holo-IscR. Furthermore, as H_2_O_2_ destabilizes the Fe-S cluster of holo-IscR, activation of the *IscR-H* node in the model can only happen if the *H_2_O_2_* node is inactive. Thus, the activation of the node *IscR-H* is mathematically described as:

-
${\rm IscR} - {\rm H } = 1\,\, {\rm if\,\, IscR} - {\rm A} \wedge {\rm Isc} \wedge \neg {H}_2{O}_2$

**Table 2.** **Logical rules associated with each node of the model**. The Variable column gives if the associated variable to each node (ternary or Boolean). For each level (Target value column) is given the logical rule to be satisfied (Logical rule column) except for the input nodes for which the initial values are fixed during the simulations.

Node	Variable	Target value	Logical rule
*Feext*	Tern.	NA	Input
*O*_2_	Tern.	NA	Input
*Fefree*	Tern.	1	(¬*Fe_ext_* ∧ ((*H*_2_*O*_2_ ∧ ¬*OxyR*) ∨ *RyhB* = 2)) ∨
			((*Fe_ext_*= 2 ∨ (*Fe_ext_*= 1 ∧ ¬*O*_2_)) ∧ *Fur* = 2 ∧ *RyhB <* 2)) ∨
			(*Fe_ext_*= 1 ∧ *O*_2_ ≥ 1 ∧ *RyhB <* 2 ∧ ¬(*H*_2_*O*_2_ ∧ *Fur <* 2))
		2	((*Fe_ext_*= 2 ∨ (*Fe_ext_*= 1 ∧ ¬*O*_2_)) ∧ (*Fur <* 2 ∨ (*RyhB* = 2 ∧ *Fur* = 2)))
			∨ (*Fe_ext_*= 1 ∧ *O*_2_ ≥ 1 ∧ (*RyhB* = 2 ∨ (*H*_2_*O*_2_ ∧ *Fur <* 2)))
*H_2_O_2_*	Bool.	1	(*O_2_*= 1 ∧¬*Hpx* ∧ *Fe_free_*= 2) ∨ (*O_2_*= 2 ∧¬*Hpx* = 2)
*OxyR*	Bool.	1	*H_2_O_2_*
*Hpx*	Tern.	1	¬*OxyR*
		2	*OxyR*
*Fur*	Tern.	1	*Fefree* = 1 ∧ (¬*H_2_O_2_* ∨ *OxyR*)
		2	*Fefree* = 2 ∧ (¬*H_2_O_2_* ∨ *OxyR*)
*RyhB*	Tern.	1	*Fur* = 1
		2	¬*Fur*
*IscR*-*A*	Bool.	1	¬*IscR*-*H*
*IscR*-*H*	Bool.	1	*IscR*-*A*∧ *Isc* ∧¬*H_2_O_2_*
*Isc*	Bool.	1	¬*IscR*-*H*∧¬*RyhB* ∧ *Fe_free_*= 2 ∧¬*H_2_O_2_*
*Suf*	Tern.	1	¬*Fur* = 2 ∧ (*OxyR* ∨ *IscR*-*A*)
		2	¬*Fur* = 2 ∧ *OxyR* ∧ *IscR*-*A*
*ErpA*	Bool.	1	¬*IscR*-*H*∧¬*RyhB* = 2
*NfuA*	Bool.	1	¬*IscR*-*H*

A full description of the parameterization of each node shown in Table 2 is given in the Material and Methods section. The annotation of each node and edge, associated with bibliographical references and documentation, are included in the GINsim model file (cf Materials and Methods and the ginml file).

### The model reveals five qualitative asymptotical behaviors depending on environmental constraints

We performed simulations of the whole model and characterized the attractors (set of states reached by the system for each combination of inputs) for each of the nine environmental conditions (Table 1 for mathematical definitions). Each combination of input values determines a unique attractor. A complete description of the attractors is provided in Table S2. The model was validated by comparing predictions of the model to literature data that was not used for model construction (Table S3).

The attractor is a stable state when both input nodes values *Fe_ext_*and *O*_2_ are fixed to 1; the 8 others combinations of input values give rise to cyclic attractors. In cyclic attractors some nodes display oscillations in between their values which arise from homeostatic regulations. All homeostasis mechanisms induce such oscillations around an equilibrium. However, homeostasis oscillations are rarely observed in experimental set-up, as amplitude and period of these oscillations are too subtle and/or rapid to be determined with currently used technology.

**Figure 2.**
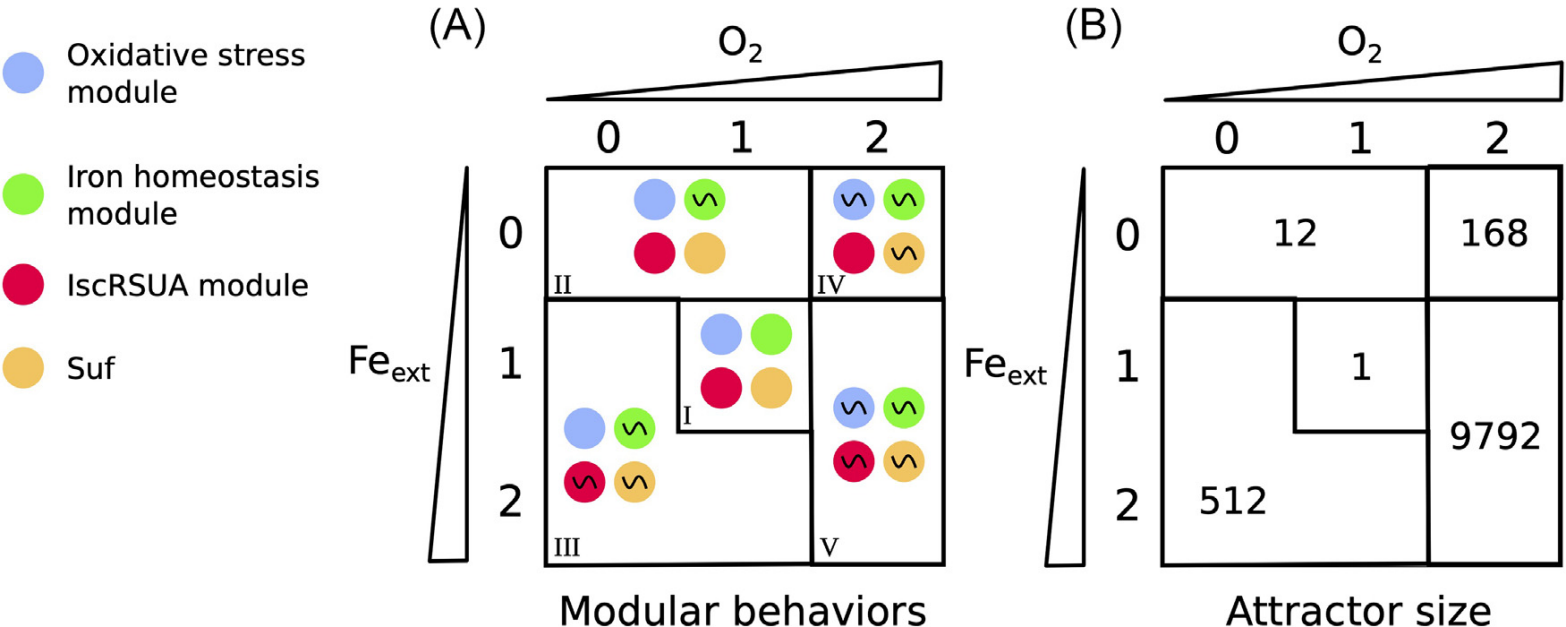
Modular description of the five asymptotic classes of the model. The two axes of the square represent the environmental conditions, set by the input nodes *O*_2_ and *Fe_ext_*; the triangles are used to depict values of the inputs, from low to high (i.e. 0 to 2). The grid separates the five different classes of asymptotical behaviors of the system. **(A)**: Each area contains four circles representing the behavior of the four modules of the RG identified by a color: oxidative stress response (blue), iron homeostasis module (green), IscRSUA module (red), and the *Suf* node (yellow). The symbol ∼ denotes an oscillatory asymptotical behavior of the module, otherwise it is stable. (**B)**: The number indicates the size of the cyclical attractor (number of states) for each different class of behavior represented in A.

Subsets of nodes that systematically displayed similar behaviors emerged and were thus gathered in so-called modules (Fig. 2A). There is a clear correspondence between the modules and the biological processes, as highlighted in Fig. 1: 1) the Fe-S cluster biogenesis module containing nodes *Isc* and *IscR*; 2) the iron homeostasis module containing nodes *Fe_free_, Fur* and *RyhB* and 3) the oxidative stress module with nodes *H_2_O_2_, OxyR* and *Hpx*. Nodes *NfuA, ErpA* and *Suf* displayed distinct behaviors and were not included in a module.

A visualization of the asymptotic activities, as generated by the model at the module scale (in terms of oscillations or stability), highlights dynamics of the regulatory processes (Fig. 2A). These modules form well-known topological motifs: a circuit for the oxidative stress module (Remy et al. 2003), a chorded circuit for the iron homeostasis module (Remy et al. 2016) and a 2 petals-flower for the IscRSUA module (Didier and Remy 2012), which are 3-components motifs constituted of negative feedback loops known to potentially control oscillations (Thomas 1981). Note that a theoretical study of these motifs helps to explain the global behavior of the system (Remy and Ruet 2008).

Five classes of behaviors emerge (numbered from I to V in Fig. 2, A), characterized by the oscillating modules: (i) the system is stable when the inputs are maintained at their intermediate level (class I); (ii) only the iron module oscillates when external iron is limiting and when there is no oxidative stress (class II); (iii) all modules oscillate, except the oxidative stress module, when iron levels are high and in absence of oxidative stress (class III); (iv) all modules oscillate, except the IscRSUA module, when iron is low and oxidative stress present (class IV); (v) all modules oscillate when iron levels are high and in presence of oxidative stress (class V).

### Modular analysis of the model highlights the global adaptation of the system to environmental constraints

**Figure 3.**
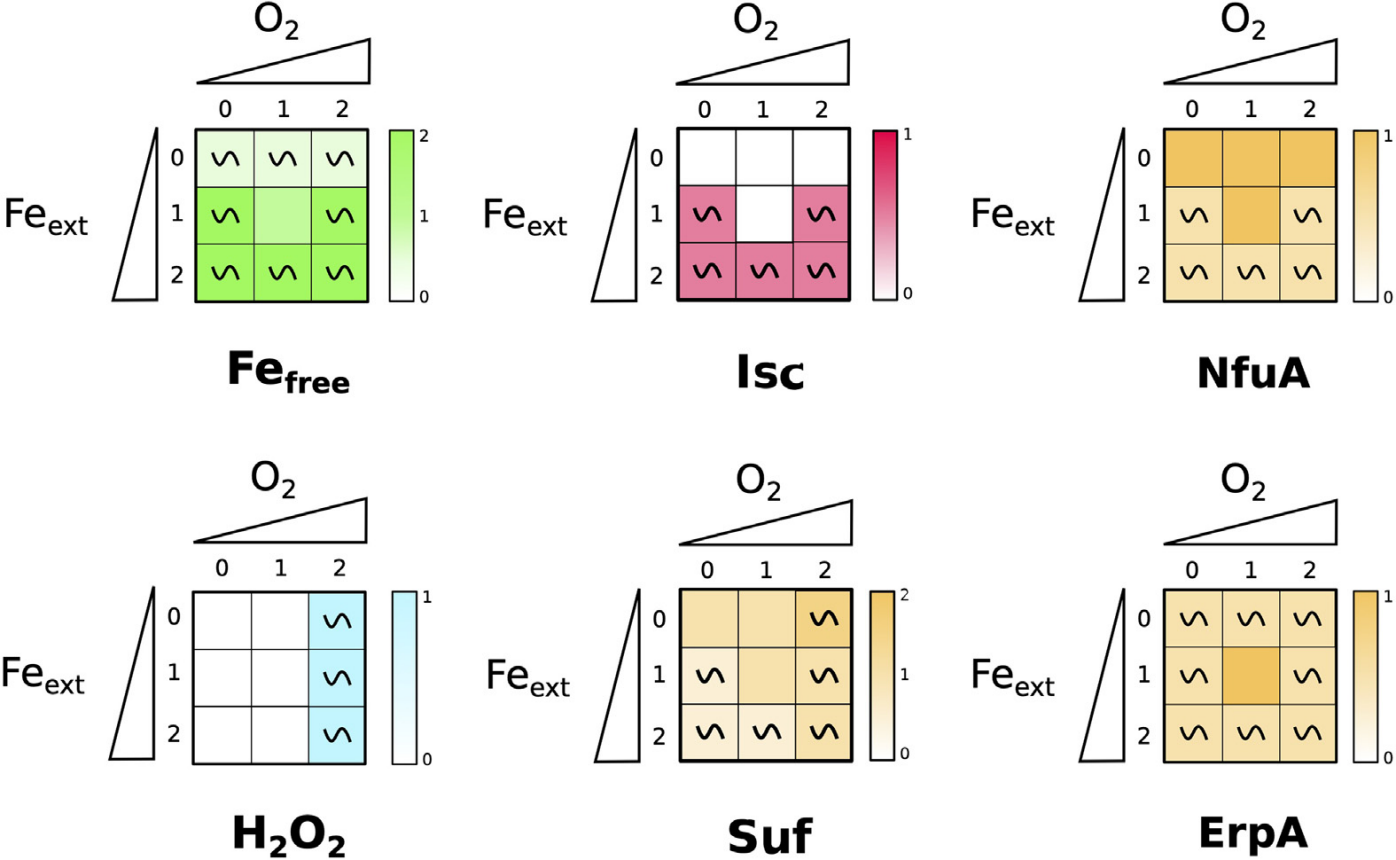
WT simulations of the model. Results of simulations for the six representative nodes in each of the 9 input combinations are shown in the form of a heatmap. The two axes of the squares represent the environmental conditions, set by the input nodes *O*_2_ and *Fe_ext_*, the triangles are used to depict values of the inputs, from low to high (i.e. 0 to 2). Each cell of the grid stands for one of the nine input conditions (depending on the values of *O_2_*and *Fe_ext_*). The qualitative asymptotical behavior of the node is indicated: oscillation (∼) or stable (no indication). Its expression level is reflected through the color graduation given by the color scale on the right of each square. In cases of oscillations, the color represents the mean of all the states of the cyclic attractor.

We leveraged the specific modular topology of the regulatory graph to analyze the adaptation to the environment. The three biological processes included in the model are represented by small strongly connected modules. Nodes *Fe_free_, H_2_O_2_*and *Isc* have been chosen as representatives of the iron homeostasis, oxidative stress, and IscRSUA modules, respectively. We also considered nodes *Suf, NfuA*, and *ErpA*, outputs of the model, to fully describe Fe-S clusters homeostasis. The asymptotic behavior of each node is displayed as a heatmap (Fig. 3).

The *Fe_free_*node is stabilized at its medium level 1 when *O_2_*= 1 and *Fe_ext_*= 1. In the other environmental conditions, the *Fe_free_* node presents oscillations tending to the value 1 (increasing between 0 and 1 in iron starvation; decreasing between 2 and 1 otherwise). This behavior reflects the need for the cell to keep its iron concentration finely tuned around an optimal concentration (*Fe_free_* = 1, which represents 10 *µ*M) that allows iron usage for biological functions while avoiding toxic accumulation in the cell.

The *H_2_O_2_*node oscillates in oxidative stress condition (*O_2_*= 2, that corresponds to presence of more than 2 *µ*M of H_2_O_2_ in the environment) regardless of the *Fe_free_*value, and is otherwise at 0 in all other situations. This behavior was expected since low doses of H_2_O_2_ are easily scavenged by catalases and peroxidases represented in the model by the Hpx node.

As expected, *Isc* and *Suf* nodes display very distinct behaviors. The *Isc* is active only in the presence of iron (and independently of O_2_), which is in agreement with biochemical data that have shown the Isc machinery to be sensitive to iron chelation (Blanc et al. 2014). *Suf* node is always present albeit at low levels (oscillating in between 0 and 1) in unstressed conditions corresponding to *Fe_ext_*= 1 and *O_2_*= 1. The model predicts that its activity increases with increasing *O_2_*and decreasing *Fe_ext_*concentrations. It reaches its maximum level in case of both iron starvation and oxidative stress. Interestingly, it should be noted that this situation is not the one with the largest attractor size (Fig. 2).

*ErpA* and *NfuA* nodes were also predicted to have distinct behaviors, which is mainly due to the fact that *RyhB* node is a regulator of *ErpA* node, but not of *NfuA* node. *NfuA* node presents a complementary behavior to *Isc* node, with *Isc* being off when *NfuA* is at its maximal values. The prediction that NfuA is more expressed when iron is limiting is in agreement with the essential role that displays this carrier in such conditions (Angelini et al. 2008, Py et al. 2018). The *ErpA* node displays oscillations in all conditions except for the *Fe_ext_*= 1 and *O_2_*= 1 situation where it is set to its maximum value 1. Both carriers are predicted to be present in all conditions, in line with their role at interacting with both machineries (Loiseau et al. 2007, Angelini et al. 2008, Py et al. 2018).

### Perturbations of the oxidative stress module affect response of the iron module but the converse is not true

To gain a better understanding of how the oxidative stress and the iron homeostasis modules interact and influence Fe-S cluster biogenesis, we simulated simple or combination of KO mutations affecting one or several modules. Such single or multiple mutants can be easily defined with the software GINsim (Materials and Methods).

**Figure 4.**
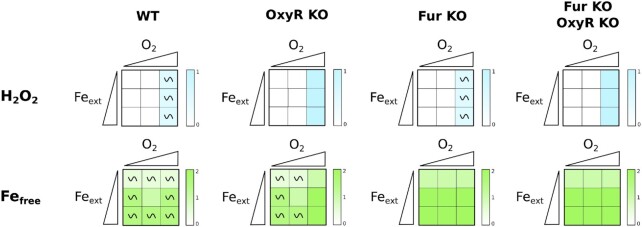
Simulations of simple and double perturbations of the iron and oxidative stress modules on nodes H_2_O_2_ and Fe_free_. Heatmap representation of the asymptotical behavior of nodes *H_2_O_2_*(top line) and *Fe_free_* (bottom line) in the nine input conditions (see caption of Fig 3) for, from left to right, WT, OxyR KO mutant, Fur KO mutant and Fur KO-OxyR KO double mutant. The two axes of the squares represent the environmental conditions, set by the input nodes *O*_2_ and *Fe_ext_*; the triangles are used to depict values of the inputs, from low to high (i.e. 0 to 2). The qualitative asymptotical behavior of the node is indicated: oscillation (∼) or stable (no indication). Node level is reflected through the color graduation given by the color scale on the right of each square. In cases of oscillations, the color represents the mean of all the states of the cyclic attractor.

We ran simulations of KO of the *OxyR* node (affecting the oxidative stress module), of the *Fur* node (affecting the iron homeostasis module), and of both. Fig. 4 shows the asymptotical behaviors of *H_2_O_2_*and *Fe_free_* nodes in the WT strain for these three perturbated situations. Expectedly, mutations affect the module to which the node belongs by blocking the oscillations. Nevertheless, it is interesting to note that the *OxyR* KO simulation, in addition to show an expected increase in *H_2_O_2_*(its production can no longer be counteracted), also displays high values of *Fe_free_* node as H_2_O_2_ attacks iron containing proteins and inactivates Fur. In contrast, while *Fur* KO simulations show an increase of *Fe_free_* in all conditions, the *H_2_O_2_* node is not affected by this perturbation, likely because catalases and peroxidases, represented by the *Hpx* node, limit H_2_O_2_ levels. We note however that, with such high levels of *Fe_free_*, even the transient production of H_2_O_2_ in the *Fur* KO mutant (see *O_2_*= 1 and *Fe_ext_* ≥ 1 in Fig. 4) will result in production of toxic ROS *via* the Fenton reaction.

Finally, the double *OxyR* and *Fur* KO displays a combination of the two behaviors of the single mutants: the *H_2_O_2_* node displayed the same behavior as in the *OxyR* KO single mutant and the *Fe_free_*node behavior was identical to that of the *Fur* KO mutant. This is not surprising as in the double mutant the homeostatic regulators of each module are deleted. We note that, *in vivo*, accumulation of H_2_O_2_ and free iron in the double *oxyR fur* mutant as predicted by the model should result in growth defects due to Fenton reaction. A similar situation has been seen in biological situations such as in the Hpx- *fur-* double mutant (Varghese et al. 2007).

### Perturbations in iron homeostasis and oxidative stress induce Suf

**Figure 5.**
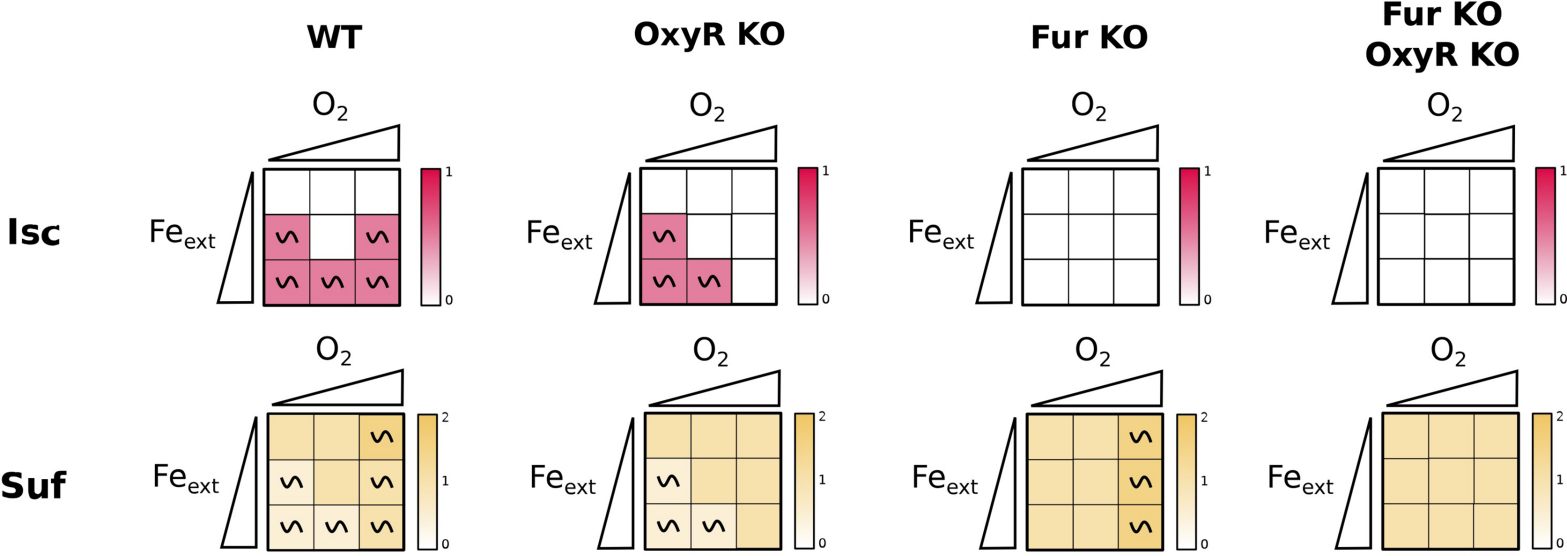
Modules perturbations effects on the Fe-S cluster biogenesis machineries. Heatmap representation of the asymptotical behaviour of nodes *Isc* (top line) and *Suf* (bottom line) in the nine input conditions (see caption of Fig 3) for, from left to right, WT, *OxyR* KO mutant, *Fur* KO mutant and *Fur* KO-*OxyR* KO double mutant. The two axes of the squares represent the environmental conditions, set by the input nodes *O*_2_ and *Fe_ext_*; the triangles are used to depict values of the inputs, from low to high (i.e. 0 to 2). The qualitative asymptotical behavior of the node is indicated: oscillation (∼) or stable (no indication). Node level is reflected through the color graduation given by the color scale on the right of each square. In cases of oscillations, the color represents the mean of all the states of the cyclic attractor.

We next examined the effect of perturbing the oxidative and/or the iron homeostasis module on Fe-S cluster biogenesis by looking at the *Isc* and *Suf* nodes (Fig. 5). Simulations with the *OxyR* KO mutant show that this mutation affects both *Isc* and *Suf* behaviors when *O_2_*= 2. Indeed, *Isc* expression is predicted to be turned off in this situation (owing to continuous ROS production, Fig. 4), leaving only the *Suf* node present, albeit at diminished levels as compared to the WT situation (*Suf* = 1 for any *Fe_free_*value when *O_2_*= 2). Strikingly, simulations with *Fur* KO mutant show that *Isc* activity is completely turned down in all situations (due to the constant *RyhB* repression on the *Isc* node), while *Suf* expression is predicted to be turned up in this case. Perturbing both modules retained these features with *Isc* node turned off (*Isc* = 0) and *Suf* node stably turned on (*Suf* = 1) in all situations. In conclusion, perturbation of either module decreases activity of *Isc* leaving *Suf* as the functional Fe-S cluster biogenesis system, in agreement with the accepted role of Suf as the only stress response system.

**Figure 6.**
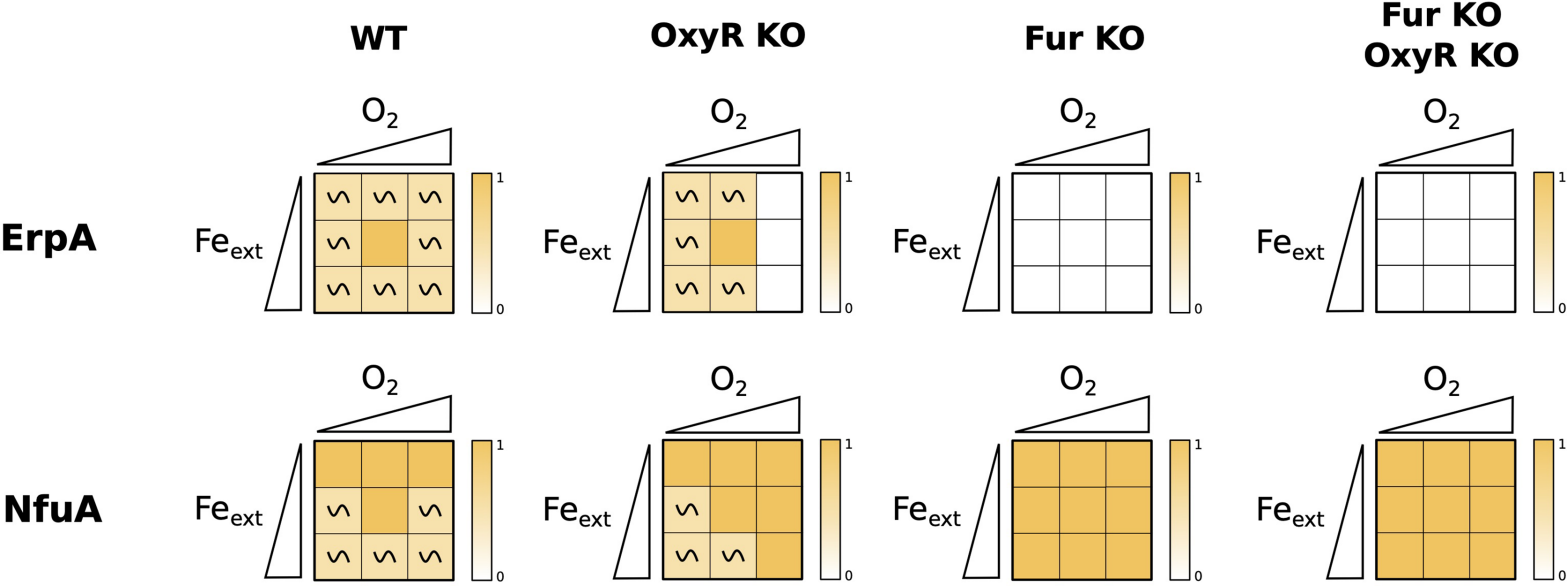
Modules perturbations effects on the ATCs. Heatmap representation of the main nodes depending of *Fe_ext_*and *O*_2_. The two axes of the squares represent the environmental conditions, set by the input nodes *O*_2_ and *Fe_ext_*; the triangles are used to depict values of the inputs, from low to high (i.e. 0 to 2). The qualitative asymptotical behavior of the node is indicated: oscillation (∼) or stable (no indication). Node level is reflected through the color graduation given by the color scale on the right of each square. In cases of oscillations, the color represents the mean of all the states of the cyclic attractor.

*ErpA* and *NfuA* nodes, representing the ATCs, were diametrically affected in the *OxyR* and *Fur* KO simulations (Fig. 6). The *ErpA* node was turned off (*ErpA* = 0) when *O*_2_ = 2 in the *OxyR* KO mutant, and even more strikingly it was predicted to be off in all conditions in the *Fur* KO mutant. In sharp contrast, the *NfuA* node was constitutively on (*NfuA* = 1) in the same *OxyR Fur* KO simulated mutant. Thus, the model further indicates that NfuA is mainly a stress response ATC as opposed to ErpA which expression is predicted to be turned down in response to stress perturbations.

### Model predicts growth defects situations

Deficiencies in Fe-S cluster biogenesis are known to cause growth defects. We thus used the model to predict KO mutants for which the *Isc* and *Suf* nodes would be turned off at particular input *O_2_* and *Fe_ext_*values. The prediction is that such mutants should exhibit growth defects in these environmental conditions as Fe-S cluster biogenesis cannot be fully ensured. Out of 54 simple mutants, only the *IscR-A* KO and the *Suf* KO mutants displayed situations in which both *Isc* and *Suf* nodes equaled 0. For combinations of double mutants, only combinations of mutants including *IscR-A* KO or *Suf* KO mutation displayed situations for which nodes *Isc* and *Suf* were both turned down to 0. The ensemble of these data is available online (https://gitlab.com/Laurent.Tichit/fe-s-cluster-biogenesis-logical-model/tree/master/Results/S4_Fig).

**Figure 7.**
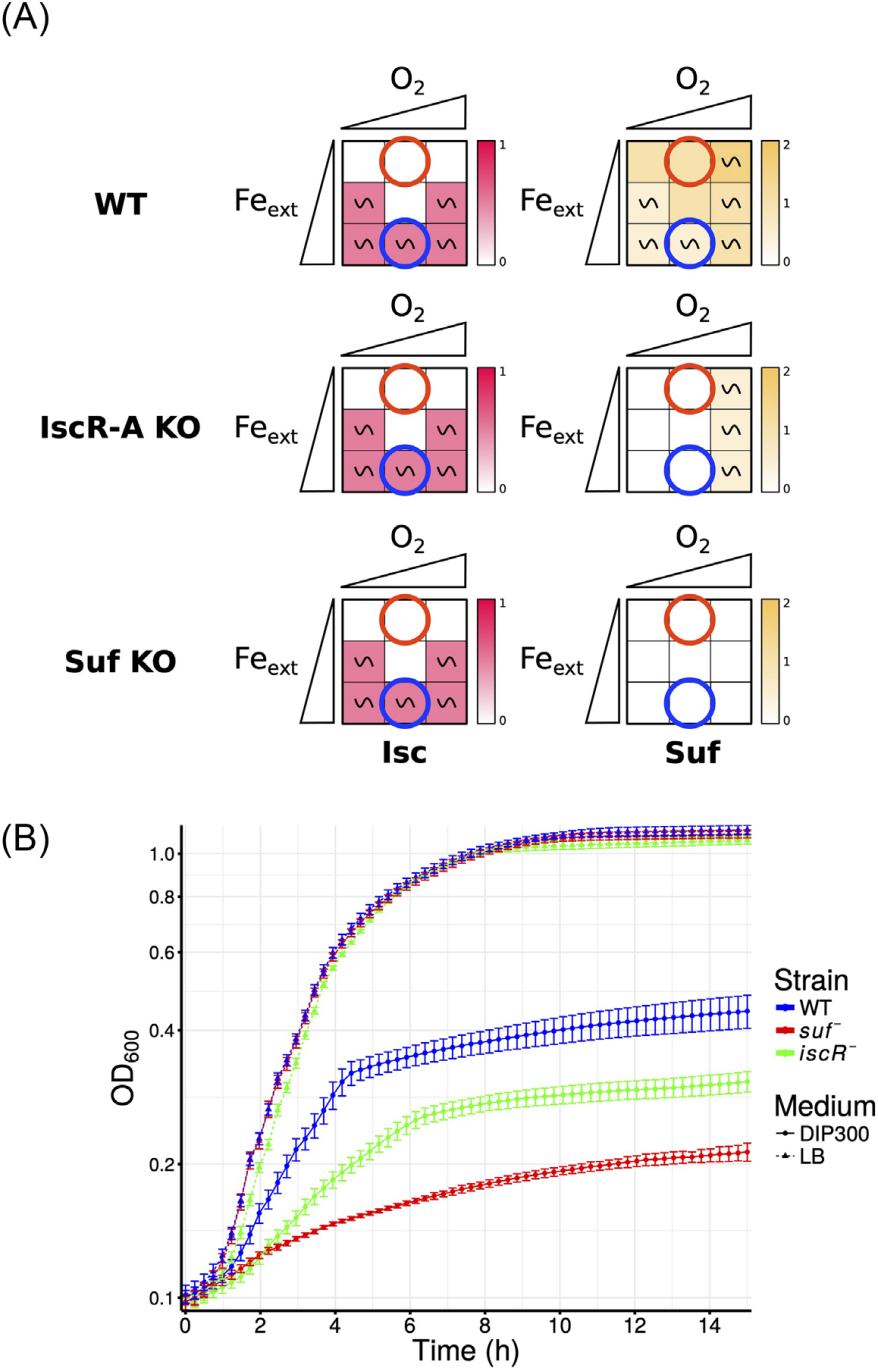
Growth defects predictions and experimental verification. **(A)** Heatmap representation of situations where neither the Isc and Suf machineries are active in the model. The two axes of the squares represent the environmental conditions, set by the input nodes *O*_2_ and *Fe_ext_*; the triangles are used to depict values of the inputs, from low to high (i.e. 0 to 2). The qualitative asymptotical behavior of the node is indicated: oscillation (∼) or stable (no indication). Node level is reflected through the color graduation given by the color scale on the right of each square. In cases of oscillations, the color represents the mean over all the states of the cyclic attractor. Red and blue circles correspond to the experimentally tested conditions (iron starvation and iron replete conditions respectively). **(B)** Experimental verification of the predictions. The dashed and filled lines correspond respectively to iron replete and iron deficient conditions, respectively. Iron starvation was induced using 300 *µ*M of dipyridyl chelator. Growth was measured at OD_600_ for 14 h at 37°C using a TECAN microplate reader.

The model predicts an lack of Fe-S cluster biogenesis system in the *Suf* KO mutant when *Fe_ext_* = 0, in agreement with the fact that *E. coli suf* mutants are sensitive to iron starvation (Outten et al. 2004) (Fig. 7 A bottom panel). Interestingly, the model predicts deficiency in Fe-S cluster biogenesis during iron starvation conditions for the *IscR-A* KO mutant. We thus tested if an *iscR* mutant displayed growth deficiencies in iron limiting conditions by growing *E. coli* WT or the *iscR* mutant in medium treated or not with dipyridyl (Dip), a well-known iron chelator (Fig. 7 B and C). A *suf* mutant strain, deleted for the whole *suf* operon, was also included as a control. Both *iscR* and *suf* mutants grew normally in rich medium (Fig. 7B). As expected, adding 300 *µ*M of Dip iron chelator slightly affected growth of the WT strain as iron is needed for many cellular processes. However, as predicted by the model, this growth defect phenotype was aggravated in the *iscR* and the *suf* mutants, thus validating the model prediction that the *iscR* mutant presents growth defects during iron starvation due to its inability to make Fe-S clusters.

## Conclusion

We have presented here a logical model that enables us to describe for the first time in details how Fe-S cluster biogenesis is finely controlled in response to iron availability and oxidative conditions. It is based on the integration of biological data from literature and genetic experiments that were translated in logical rules and regulatory graph. The simulations of the model recapitulate qualitatively the possible behaviors of the system depending on iron and oxygen availability. We have proposed a visualization of these behaviors that facilitates the detection of changes with the environmental conditions and the different perturbations of the model.

Our model relies on the logical formalism, a discrete mathematical framework that has proved to be particularly successful to understand dynamical features of complex biological processes, and to model and analyze regulatory and signaling networks.

Previous models based on ordinary differential equations were built in order to analyze iron homeostasis in *E. coli*, and reported damped oscillations of Fur regulated gene expression (Amir et al. 2010). In another modeling driven study, Fe-S cluster biogenesis was predicted to compensate iron import in order to avoid iron toxicity at high iron concentrations (Semsey et al. 2006). Other models were built in order to analyze oxidative stress in *E. coli*, either for understanding the contributions of AhpCF, KatG and the H_2_O_2_ diffusion in the membrane (Uhl and Dukan 2016), or the effects of carbon deprivation on oxidative stress response (Adolfsen and Brynildsen 2015). However, the crucial impact of oxidative conditions in coordination with iron concentration in order to modulate Fe-S cluster biogenesis had not been integrated in the modeling process.

Logical (and more generally discrete) modeling is limited to qualitative results, such as presence or absence of a component in given environmental conditions. Despite this coarse-grained abstraction, such a qualitative model captures the most salient properties of the modeled systems. Here, it allowed us to summarize the classes of qualitative behaviors depending on environment conditions at the scale of biological processes (modules). We thereby highlight essential features of Fe-S cluster biogenesis. The modular architecture of the network is interesting and could be further exploited. Indeed, it is believed that the network architecture can help control the network dynamics to some extent. Several methods also attempt to decompose the large network into smaller modules in order to analyze them and then recompose the overall dynamics. This model provides an excellent example for working through these methodological challenges.

Analysis of our model reinforces the classical paradigm of Isc being the homeostatic machinery expressed in favorable conditions in opposition to Suf as the stress responsive machinery (Fig. 3). Indeed, perturbing either the oxidative stress or the iron homeostasis responses is predicted to generally induce expression of Suf and to turn down Isc. However, the analysis of our model highlights interesting complexities.

Our model predicts that expression of the Isc machinery is correlated with iron availability but independent of oxidative conditions (Fig. 3). Thus, surprisingly, Isc is predicted to be present during oxidative stress. In fact, expression of Isc has been observed in cells experiencing protracted exposure to H_2_O_2_, validating our model prediction, although Isc was shown to be not functional for maturation of Fe-S cluster containing targets in such conditions (Jang and Imlay 2010). Note that a similar observation was carried out using phenazine metho sulfate, another oxidative stress causing chemical damages (Gerstel et al. 2020). One explanation to this somewhat paradoxical behavior may be that one or multiple proteins of the Isc machinery serves other functions than Fe-S cluster biogenesis in presence of oxidative stress. IscS in particular also functions as a cysteine desulfurase for tRNA modifications, an activity that may not be compromised in presence of ROS and that cannot be replaced by SufS (Bühning, 2017).

The analysis of the attractors of our model raises interesting observations. The size of the attractors -i.e. the number of states they contain- correlates with the number of oscillating modules and the amplitude of the oscillations in case of multilevel variables (Fig. 2B). Thus, the larger the size of the attractor, the higher the amplitude of the oscillations. In biological terms, the size of the attractor can thus be interpreted as proportional to the biological activity needed to maintain equilibrium. Somewhat counter-intuitively, the combination of two environmental stresses, e.g. iron starvation and oxidative stress (class IV), does not induce an attractor size larger, and thus does not imply more biological activity to cope with, than when applying only one, e.g. oxidative stress. In the same vein, the situation that gives rise to Suf highest expression in our model is not the one that is predicted to be the more destabilizing for the ensemble of the system (Fig. 2). These results suggest that while iron starvation diminishes the burden of oxidative stress by hindering Fenton reaction, it worsens Fe-S cluster biogenesis as it becomes even more difficult to synthesize Fe-S clusters in presence of ROS and in iron starved conditions, rising the need for the Suf machinery.

Interestingly, our model also gives new insights on ATC transporter usage in function of environmental conditions. The model predicts that NfuA expression is anti-correlated with that of Isc, and becomes higher in stress conditions or by perturbing the iron homeostasis or oxidative response modules (Fig. 3 and Fig. 6). Conversely, ErpA is predicted to be expressed in almost all conditions in the WT, with a peak at medium iron and oxygen levels, but to be turned down when input modules are perturbed by KO mutations (Fig. 3 and Fig. 6). Thus, our model highlights the role of NfuA as a stress response carrier while ErpA behaves as an housekeeping component of Fe-S cluster biogenesis, in a parallel situation to Suf and Isc. This model is in agreement with several observations in the literature: in particular, an *nfuA* mutant has been shown to be required during oxidative stress and iron starvation conditions (Angelini et al. 2008) while *erpA* is essential in normal conditions, as would be expected for an important stress or housekeeping component, respectively. However, carriers usage is probably more versatile than that of Fe-S cluster biogenesis machineries. Indeed, both NfuA and ErpA have been shown to be together required for maturation of a subset of Fe-S cluster containing protein targets and to be able to interact with either the Suf or Isc machineries (Py et al. 2018), and this is reflected in our model by the fact that both carriers are expressed together in all conditions in the WT.

Cells can be exposed to H_2_O_2_ even under anaerobic conditions, in particular during infection (Imlay 2019). ‘Anaerobic oxidative stress’ is unfortunately insufficiently documented, especially in terms of genetic responses, and we could not integrate this particular case in our model. However, there are no reasons to think that oxidation of OxyR by H_2_O_2_ should occur differently anaerobically than in presence of oxygen. In line with this, a study identified that a comparable set of genes from the OxyR regulon were also induced in both aerobic and anaerobic conditions, albeit to a greater extent anaerobically (Kang et al. 2013). Nitrogen metabolism and respiratory pathways were however differentially regulated. Hence, a proxy for what may happen for H_2_O_2_ stress in anaerobic conditions may simply be to use our model with inputs O_2_ = 2 (H_2_O_2_ stress). In the long term, a perspective of our work will thus be to refine our model in order to include this particular condition.

Finally, in the past few years Fe-S cluster biogenesis machineries usage has been shown to have important consequences on the sensitivity to antibiotics, such as aminoglycosides and fluoroquinolones (Ezraty et al. 2013, Gerstel et al. 2020). Aminoglycosides enter the cell through the proton motive force generated by respiratory complexes I and II (Nuo and Sdh), which contain numerous iron-sulfur containing proteins. Therefore, complexes I and II activity is dependent on Fe-S cluster biogenesis, specially Isc which is most potent to mature them. As a consequence, iron starvation, in which Suf is used instead of Isc, decreases susceptibility to aminoglycosides, and RyhB has a major role in promoting this resistance phenotype through its inhibition of Isc synthesis (Chareyre et al. 2019). Thus, by enabling to precisely predict machineries usage in function of the environmental conditions, our model should also predict situations for which the bacterium becomes resistant to aminoglycosides. For instance, the Fur mutant is predicted to be resistant to aminoglycosides regardless of the growth conditions, as Isc is down-regulated (Fig. 5), a phenotype that has been validated in laboratory growth conditions (Ezraty et al. 2013).

## Materials and methods

### Logical modeling

We chose to work within the logical framework which offers a qualitative description of the molecular mechanisms, and is therefore well suited to elucidate main processes of the cell functioning and to pinpoint key biological components controlling the modelled process. It relies on a Regulatory Graph (RG), a signed labelled directed graph whose nodes represent the biochemical species, and directed signed edges linking pairs of nodes represent the regulations (activations or inhibitions). The selection of molecular players to be included in the RG and their mutual interactions are based on an extensive analysis of the literature and on our current understanding of the molecular mechanisms controlling Fe-S cluster biogenesis. To complete this model description and obtain a dynamical and predictive model, we attached to each node a discrete variable representing its activity level that reflects the node's ability to regulate its targets in the network. With a Boolean variable, the component is active (on/1) or inactive (off/0). In some situations, more than two levels are necessary to distinguish different regulatory roles of the component in the network and the variable is multilevel. The threshold at which a regulation takes place is indicated as a label on the edge (1 by default). Vectors encompassing values for all components are called logical states. The impacts of combinations of regulators on the state of a component are encoded through logical rules expressed with logical operators AND, OR, NOT (Thomas 1973).

Given a logical state, the logical rules determine which components are able to change their value. We use an asynchronous dynamic: only one node can update at a time. Hence, two successive states differ by only one component, and a state has as many successors as the number of components called to update. This dynamic is thus non deterministic, and all the possible asynchronous trajectories can be represented in the State Transition Graph (STG), a directed graph whose nodes are the logical states of the network, and edges connect successive states.

To characterize the long-time behaviors of the system, we identify the attractors of the model which are the terminal strongly connected components (SCC) of the STG, i.e. the sets of states in which the dynamics is trapped. Their gene expression patterns reveal the activities of components at these asymptotic regimes. We distinguish two types of attractors, the stable states constituted of a unique node (all the components are stable); and the complex (or cyclical) attractors containing more than 2 nodes (some components display oscillations). The identification of complex attractors is often computationally difficult because of the combinatorial explosion of the STG. We take advantage of functionalities implemented in GINsim, in particular we generate the Hierarchical Transition Graph (HTG) that is a compaction of the STG (Bérenguier et al. 2013).

### Full description of the logical model construction

We use the following notations for the logical operators: ∧: AND; ∨: OR; ¬: NOT. We describe the rules only for non-negative levels of the variables, which means that, by default, the variable takes the value 0.

#### Fe_ext_

The *Fe_ext_* node describes the extracellular iron levels met by *E. coli*. The associated variable *Fe_ext_*has 3 levels: level 0 corresponds to an external iron concentration lower than 1 µM (starvation); level 1 to a concentration between 1 and 5 µM (Hartmann and Braun 1981), and level 2 to a concentration greater than 5–10 µM (Andrews et al. 2003; Klebba et al. 1982).

#### O_2_

The *O_2_* node describes oxygen levels. The associated variable *O*_2_ has 3 levels: level 0 corresponds to anaerobiosis; level 1 to aerobiosis; and level 2 to oxidative stress with external [H_2_O_2_] *>* 2 µM. Indeed, the latter condition corresponds to external [H_2_O_2_] required to activate OxyR transcription factor (Li and Imlay 2019).

#### H_2_O_2_

The *H_2_O_2_* node describes the intracellular H_2_O_2_ concentration. The associated variable *H*_2_*O*_2_ is Boolean: level 0 corresponds to an internal [H_2_O_2_] *<* 50 nM, and level 1 to the intracellular [H_2_O_2_] ≥ 50 nM (oxidative stress), which allows the activation of the OxyR transcription factor. The *H_2_O_2_* node is activated by nodes *O_2_*and *Fe_free_*, and inhibited by *Hpx* (*ie*. KatG and AhpCF). Basal Hpx expression levels limit H_2_O_2_ accumulation under aerobic conditions, yet are not sufficient under oxidative stress conditions. Furthermore, iron accumulation can lead to oxidative stress through iron oxidization (Imlay 2013, Khademian and Imlay 2017, Park and Imlay 2003; Imlay 2019). Thus:


\begin{eqnarray*} {H}_2{O}_2 = 1\,\,{\rm if}\,\,\left( {{O}_2 = 1 \wedge \neg Hpx \wedge F{e}_{free} = 2} \right) \vee \left( {{O}_2 = 2{\rm{ }} \wedge Hpx{\rm{ }} < 2} \right) \end{eqnarray*}

#### OxyR

The *OxyR* node describes OxyR transcription factor activity. As OxyR is activated if [H_2_O_2_] ≥ 50 nM (Seaver and Imlay 2001b; Imlay 2019), we use a Boolean variable with *OxyR* = 1 corresponding to OxyR activation.


\begin{eqnarray*} OxyR = 1\,\,{\rm if}\,\,{H}_2{O}_2 \end{eqnarray*}

#### Hpx

The *Hpx* node corresponds to the H_2_O_2_ scavengers KatG and AhpCF expression levels. We did not include KatE as its contribution in H_2_O_2_ response has been shown as negligible (Seaver and Imlay 2001a). KatG and Ahp are both activated by OxyR (Seaver and Imlay 2001b, Imlay 2019). The *Hpx* variable has 3 levels: *Hpx* = 1 represents the basal expression level of KatG and Ahp, and *Hpx* = 2 their OxyR-induced expression level. *Hpx* = 0 is used only to represent a *Hpx* mutant. Thus:

-
$Hpx = 2\,\, {\rm if}\,\, OxyR$

-
$Hpx = 1\,\, {\rm if}\,\, \neg OxyR$

#### Fe_free_

The Fe_free_ node describes the free internal iron concentrations and has 3 levels: *Fe_free_*= 2 corresponds to concentration levels ≥ 50 µ*M* [Fe^2+^]; *Fe_free_*= 1 to concentration levels around 10 µM [Fe^2+^] (or 150 µM dipyridyl); and *Fe_free_* = 0 to concentration levels ≤ 1 µM [Fe^2+^] (or 300 µM dipyridyl) (Keyer and Imlay 1996, Beauchene et al. 2017; Wofford et al. 2019). Fe_free_ depends on iron import systems regulated by Fur (Hantke 1981, Bagg and Neilands 1987). Oxygen limits iron availability (Beauchene et al. 2015; Beauchene et al. 2017; Wofford et al. 2019). During iron starvation RyhB raises [Fe_free_] by inducing a sparing response (Massé and Gottesman 2002, Prévost et al. 2007). During oxidative stress OxyR limits [Fe_free_] through the Dps storage protein activity (Park et al. 2005). Moreover, the accumulation of intracellular iron in the double mutant *fur- Hpx-* (Varghese et al. 2007) led us to postulate that Fe_free_ levels would raise if both OxyR and RyhB are present. Thus:

#### Fur

The *Fur* node represents Fur repression activity. The *Fur* variable has 3 levels: *Fur* = 0 corresponds to inactive Fur; *Fur* = 1 to Fur-Fe levels allowing partial RyhB repression (see below); and *Fur* = 2 to Fur-Fe levels allowing full repression of all its regulated genes. The Fur node is inhibited by H_2_O_2_, and activated by nodes *Fe_free_* and *OxyR*, as OxyR is required to maintain Fur repression activity (Varghese et al. 2007). Thus:

-
$Fur = 2\,\, {\rm if}\,\, Fe_{free} = 2 \wedge ( {\neg H_2O_2 \vee OxyR} ),$

-
$Fur = 1\,\, {\rm if}\,\, Fe_{rm free} = 1 \wedge ( {\neg H_2O_2 \vee OxyR} )$

#### RyhB

The *RyhB* node describes RyhB expression levels and has 3 levels: level 0 corresponds to RyhB repression under iron replete conditions, level 1 to expression levels which inhibit Isc translation, and level 2 to expression levels inhibiting both Isc and ErpA (Mandin et al. 2016). We postulated that the Isc and ErpA inhibitions present different and ordered regulatory thresholds, based on the differential regulation of *erpA* by RyhB as compared to *iscSUA* (Mandin et al. 2016; Desnoyers et al. 2009). As RyhB is only regulated by Fur, the logical rules describe the following:

-
$RyhB = 2\,\, {\rm if}\,\, \neg Fur$

-
$RyhB = 1\,\, {\rm if}\,\, Fur = 1$

#### IscR

We decomposed the IscR transcription factor in two boolean nodes, *IscR-A*, and *IscR-H*, corresponding to the apo- and holo- forms of IscR, respectively, in order to describe properly the IscR regulation and maturation. Indeed holo-IscR represses both the Isc machinery and its own expression (Schwartz et al. 2001) while apo-IscR activates Suf expression (Giel et al. 2006). We considered IscR maturation to be only Isc-dependent (Vinella et al. 2013, Mettert and Kiley 2014). Indeed Suf-dependent IscR maturation only occurs biologically in the Isc mutant (Mettert and Kiley 2014), and is not sufficient to restore IscR full repression activity (Vinella et al. 2013).

Thus:

-
$IscR - H = 1\,\, {\rm if}\,\, IscR - A \wedge Isc \wedge \neg H_2O_2$

-
$IscR - A = 1\,\, {\rm if}\,\, IscR - H = 1$

#### Isc

The node *Isc* describes the Isc machinery activity. The *Isc* variable is boolean: *Isc* = 0 corresponds to the absence of Isc activity, and *Isc* = 1 corresponds to active Isc. Isc expression is repressed by IscR-Holo and by RyhB, and H_2_O_2_ inhibits Isc machinery activity (Schwartz et al. 2001, Desnoyers et al. 2009, Jang and Imlay 2010). Conversely Free iron is needed for Isc activity. Thus:

-
$Isc = 1\,\, {\rm if}\,\,\neg IscR - H \wedge \neg RyhB \wedge {Fe}_{free} = 2 \wedge \neg {H}_2{O}_2$

#### Suf

The node *Suf* describes *sufABCDSE* expression levels. The *Suf* variable has 3 levels: level 0 corresponds to absence of Suf, level 1 to medium expression levels, and level 2 to high expression levels. Suf is activated by OxyR and IscR-A, and repressed by Fur (Yeo et al. 2006, Lee et al. 2008). Thus:

-
$Suf = 2\,\, {\rm if}\,\, Fur < 2 \wedge OxyR \wedge IscR - A$

-
$Suf = 1\,\, {\rm if}\,\, Fur < 2 \wedge ( {OxyR \vee IscR - A} ) \wedge \neg ( {OxyR \wedge IscR - A} )$

#### ErpA

The node *ErpA* describes *erpA* expression levels. The *ErpA* variable is boolean, where *ErpA* = 0 corresponds to low expression levels, and *ErpA* = 1 to high expression levels. ErpA is inhibited by IscR-H and RyhB when its levels are high (see above) (Giel et al. 2006, Mandin et al. 2016). Thus:

-
$ErpA = 1\,\, {\rm if}\,\, \neg IscR - H \wedge RyhB < 2$

#### NfuA

The node *NfuA* describes *nfuA* expression levels. The *NfuA* variable is boolean, where level 0 corresponds to low expression levels, and level 1 to high expression levels. NfuA is inhibited by IscR-H (Giel et al. 2006). Thus:

-
$NfuA = 1\,\,{\rm if}\,\,\neg IscR - H$

### Logical modeling Software GINsim

We used the freely available software GINsim (Gene Interaction Network simulation, http://ginsim.org) for the edition, analysis and simulation of the regulatory graph (Chaouiya et al. 2012). Once the model defined, several simulation tools are proposed to simulate the State Transition Graph, the Hierarchical Transition Graph, do reduction of model or compute the stable states. GINsim also enables the simulation of mutants (loss-of-function, ectopic gene expression, and combinations) by blocking the levels of expression of the corresponding variables. To further ease the analysis of multiple perturbations, we have written a set of scripts in Python and R, which iteratively compute the behavior of our model for all input values and mutants considered, and generate heatmaps representing their behavior.

### Growth assays

Sensitivity to iron starvation was measured as follows: overnight stationary phase culture grown in LB media were diluted 1:500 in 3 mL LB without or with 300 *µ*M DIP (2,2’ bipyridyl, Sigma-Aldrich, ≥ 99% purity, CAS 366–18-7). Cells were grown with shaking (300 rpm) at 37°C for 9 hours, before being diluted 1:100 in a 96 well plate containing either LB with or without 300 *µ*M DIP. Growth of the different strains were measured by reading the OD_600_ of each well during 14 h at 37°C with shaking at 330 rpm on a TECAN Spark 510 M as in (Chareyre et al. 2019).

### Reproducibility of the results

In order to allow reproducing the results, we have developed an application allowing to:

1. load the logical model (in the GINsim format) corresponding to the current study
2. after choosing N nodes of interest, build a set of mutant models (which contains the ’wild type’ and all the single mutants, double mutants, …, N-uple mutants)
3. compute the dynamics of each model using GINsim
4. choose the readouts (the variables of interest representing either the expression level, activity or discrete concentrations) to visualize, and optionally search for mutants which may lead to the desired phenotype (e.g. by default, low levels of Isc activity and Suf expression in the attractor)
5. finally, generate the heatmap representation of the asymptotic behaviors of the readouts for each mutant.

This application is installable directly from source from gitlab and can be run via executing a docker file containing all the required dependencies. It is available at https://gitlab.com/Laurent.Tichit/fe-s-cluster-biogenesis-logical-model/.

## Supplementary Material

uqad003_Supplemental_File
