# Supplementary material for: Analysis of a logical regulatory network reveals how Fe-S cluster biogenesis is controlled in the face of stress
Source: Microlife. 2023 Mar 2;4:uqad003. doi: 10.1093/femsml/uqad003 (PMC10117729; doi:10.1093/femsml/uqad003)
Supplement: uqad003_Supplemental_File [file uqad003_supplemental_file.pdf]

S1 Table - Description of the variables

Node	Node description	Levels	Biological description	References
Fe_{ext}	External iron concentrations	0	Low [Fe2+]ext ($[Fe^{2+}]_{ext} \leq 1\mu M$)	[Andrews et al., 2003] [Klebba et al., 1982] [Hartmann and Braun, 1981]
		1	Medium $[Fe^{2+}]_{ext}$ ($1\mu M \leq [Fe^{2+}]_{ext} < 10\mu M$)	
		2	High [Fe2+]ext ($10\mu M \leq [Fe^{2+}]_{ext}$)	
O_2	Oxygen levels	0	Anaerobiosis	[Li and Imlay, 2018]
		1	Aerobiosis ($[H_2O_2]_{ext} \leq 2$ μM)	
		2	Oxidative stress ($[H_2O_2]_{ext} > 2$ μM)	
Fefree	Free iron concentration	0	~ 1μM [Fe2+] (= 300 μM DIP)	[Beauchene et al., 2017] [Keyer and Imlay, 1996] [Wofford et al., 2018] [Chareyre and Mandin, 2018]
		1	~ 10μM [Fe2+] (= 150 μM DIP)	
		2	~ 100μM [Fe2+] (= 0 μM DIP)	
Fur	Fur-Fe repression activity	0	No activity (Apo-Fur)	[Seo et al., 2014] [Bagg and Neilands, 1987] [Beauchene et al., 2015] [Chareyre and Mandin, 2018]
		1	Low activity	
		2	High activity (Fur-Holo)	
RyhB	RyhB expression	0	Low expression levels	[Prévost et al., 2007] [Mandin et al., 2016] [Levine and Hwa, 2008]
		1	Medium expression levels	
		2	High expression levels	
Hpx	AhpCF/KatG expression	0	No expression (*ΔHpx* mutant)	[Seaver and Imlay, 2001b] [González-Flecha and Demple,
		1	Basal expression level	
		2	OxyR induced expression level	
Suf	Suf machinery expression	0	Low expression levels	[Yeo et al., 2006] [Lee et al., 2008]
		1	Medium expression levels	
		2	High expression levels	
H2O2	H_2O_2 concentration	0	[H2O2]int <50 μM	[Seaver and Imlay, 2001a]
		1	[H2O2]int ≥ 50 μM	
OxyR	OxyR transcription factor activity	0	Low activity	[Imlay, 2013]
		1	High activity	
Isc	Isc machinery activity	0	Low activity	[Desnoyers et al., 2009] [Schwartz et al., 2001] [Jang and Imlay, 2010]
		1	High activity	
IscR-A	IscR-Apo expression	0	Low expression levels	[Schwartz et al., 2001] [Vinella et al., 2013]
		1	High expression levels	
IscR-H	IscR-Holo repression activity	0	Low activity	
		1	High activity	
ErpA	ErpA expression levels	0	Low expression levels	[Giel et al., 2006] [Wright et al., 2013]
		1	High expression levels	
NfuA	NfuA expression levels	0	Low expression levels	[Angelini et al., 2008]
		1	High expression levels	

S2 Table. Description of the attractors of the model

<table>
<tr><th>Fe_{ext}</th><th>O_2</th><th>Fe_{free}</th><th>Fur</th><th>RyhB</th><th>ROS</th><th>OxyR</th><th>Cat</th><th>Suf</th></tr>
<tr><td rowspan="6">0</td><td rowspan="6">0</td><td>0</td><td>0</td><td>1</td><td rowspan="6">0</td><td rowspan="6">0</td><td rowspan="6">1</td><td rowspan="6">1</td></tr>
<tr><td>0</td><td>0</td><td>2</td></tr>
<tr><td>0</td><td>1</td><td>1</td></tr>
<tr><td>1</td><td>0</td><td>2</td></tr>
<tr><td>1</td><td>1</td><td>1</td></tr>
<tr><td>1</td><td>1</td><td>2</td></tr>
<tr><td rowspan="8">1/2</td><td rowspan="8">0</td><td>1</td><td>1</td><td>0</td><td rowspan="8">0</td><td rowspan="8">0</td><td rowspan="8">1</td><td rowspan="8">0/1</td></tr>
<tr><td>1</td><td>1</td><td>1</td></tr>
<tr><td>1</td><td>2</td><td>0</td></tr>
<tr><td>1</td><td>2</td><td>1</td></tr>
<tr><td>2</td><td>1</td><td>0</td></tr>
<tr><td>2</td><td>1</td><td>1</td></tr>
<tr><td>2</td><td>2</td><td>0</td></tr>
<tr><td>2</td><td>2</td><td>1</td></tr>
<tr><td rowspan="6">0</td><td rowspan="6">1</td><td>0</td><td>0</td><td>1</td><td rowspan="6">0</td><td rowspan="6">0</td><td rowspan="6">1</td><td rowspan="6">1</td></tr>
<tr><td>0</td><td>0</td><td>2</td></tr>
<tr><td>0</td><td>1</td><td>1</td></tr>
<tr><td>1</td><td>0</td><td>2</td></tr>
<tr><td>1</td><td>1</td><td>1</td></tr>
<tr><td>1</td><td>1</td><td>2</td></tr>
<tr><td>1</td><td>1</td><td>1</td><td>1</td><td>1</td><td>0</td><td>0</td><td>1</td><td>1</td></tr>
<tr><td rowspan="8">2</td><td rowspan="8">1</td><td>1</td><td>1</td><td>0</td><td rowspan="8">0</td><td rowspan="8">0</td><td rowspan="8">1</td><td rowspan="8">0/1</td></tr>
<tr><td>1</td><td>1</td><td>1</td></tr>
<tr><td>1</td><td>2</td><td>0</td></tr>
<tr><td>1</td><td>2</td><td>1</td></tr>
<tr><td>2</td><td>1</td><td>0</td></tr>
<tr><td>2</td><td>1</td><td>1</td></tr>
<tr><td>2</td><td>2</td><td>0</td></tr>
<tr><td>2</td><td>2</td><td>1</td></tr>
<tr><td>0</td><td>2</td><td></td><td></td><td></td><td></td><td></td><td></td><td>1</td></tr>
<tr><td>1/2</td><td>2</td><td></td><td></td><td></td><td></td><td></td><td></td><td>0/1</td></tr>
</table>

S3 Table. Model validation

Gene studied	Reference	Experimental measurements	Genetic background	Extracellular iron	Oxygen	Experimental results	Model attractor	Consistent?
oxyR							**OxyR**	
	Seaver & Imlay, 2001	*katG-lacZ [Induced by OxyR]*(U / mg of protein)	WT	LB	Aerobic	0.03 U / mg	0	y
			Ahp$^-$	LB	Aerobic	0.35 U / mg	1	
ryhB							**RyhB**	
	Baez & Shiloach, 2017	RT-qPCR (normalized to t_0)	WT	LB	Aerobic (30% Air saturation)	0.92	0 / 1	y
				LB	Aerobic (300% Air saturation)	5.15	0 / 1 / 2	
**free Fe^{2+}**							**Fe_free**	
	Salvail & al, 2010	EPR	WT	Chelex	Aerobic	20 µM	0 / 1	y
			fur$^-$*ryhB*$^-$	Chelex	Aerobic	7.5 µM	0	
		EPR	WT	Chelex (+FeSO4)	Aerobic	20 µM	1 / 2	y
			fur$^-$*ryhB*$^-$	Chelex (+FeSO4)	Aerobic	45 µM	2	
	Varghese & al, 2007	EPR	WT	LB	Aerobic	50 µM	1 / 2	**No**[a]
			Hpx-			150 µM	1 / 2	
		EPR	*Hpx-*	LB	Aerobic	150 µM	1 / 2	y
			OxyR-NI(fur)Hpx$^-$	LB	Aerobic	400 µM	2	
		EPR	*Hpx-*	LB	Aerobic	150 µM	1 / 2	y
			Hpx$^-$fur$^-$	LB	Aerobic	400 µM	2	
			OxyR-NI(fur)Hpx$^-$	LB	Aerobic	400 µM	2	

a Fur is still able to compensate the oxidative stress effects, the free intracellular iron increase is under the regulatory threshold.

Gene Studied	Reference	Experimental measurements	Genetic background	Extracellular iron	Oxygen	Experimental results	Model attractor	Consistent?
iscR							**IscR-H**	
	Vinella & al, 2013	P_{iscR}-*lacZ*	WT	LB	Aerobic	300 U / OD_{600}	0 / 1	y
				Dip = 150	Aerobic	450 U / OD_{600}	0	
	Mettert & Kiley, 2014	P_{iscR}-*lacZ*	WT	M9	Anaerobic	~ 100 Miller Units	0 / 1	**No**[b]
				M9	Aerobic	~ 1000 Miller Units	0 / 1	
		P_{iscR}-*lacZ*	WT	M9	Aerobic	~ 1000 Miller Units	0 / 1	y
			fur⁻	M9	Aerobic	~ 2000 Miller Units	0	
		P_{iscR}-*lacZ*	WT	M9	Aerobic	~ 1000 Miller Units	0 / 1	y
			fur⁻*iscSUA*⁻	M9	Aerobic	~ 2000 Miller Units	0	
erpA							**ErpA**	
	Mandin & al, 2016	P_{erpA}-*erpA-lacZ*	WT	LB	Aerobic	70 AU	0 / 1	y
				Dip = 150	Aerobic	100 AU	1	
				Dip = 300	Aerobic	60 AU	0 / 1	
		P_{erpA}-*erpA-lacZ*	*iscR*⁻	LB	Aerobic	110 AU	1	y
				Dip = 150	Aerobic	100 AU	1	
				Dip = 300	Aerobic	80 AU	0 / 1	
		P_{erpA}-*erpA-lacZ*	*ryhB-*	LB	Aerobic	60 AU	0 / 1	y
				Dip = 150	Aerobic	110 AU	1	
				Dip = 300	Aerobic	100 AU	1	
		P_{erpA}-*erpA-lacZ*	*iscR*⁻/*ryhB*⁻	LB	Aerobic	110 AU	1	y
				Dip = 150	Aerobic	120 AU	1	
				Dip = 300	Aerobic	120 AU	1	
	Giel & al, 2006	Microarray (Affymetrix values)	WT	MOPS	Anaerobic	750	0 / 1	**No**[b]
				MOPS	Aerobic	1500	0 / 1	

b The experimentally observed changes are under the regulatory threshold affecting IscR activity in our model.

Gene Studied	Reference	Experimental measurements	Genetic background	Extracellular iron	Oxygen	Experimental results	Model attractor	Consistent?
nfuA							NfuA	
	Angelini & al, 2008	RT-PCR (DIP / WT ratio)	WT	LB	Aerobic	1	0 / 1	y
			WT	Dip = 250	Aerobic	2	1	
	Giel & al, 2006	Microarray (Affymetrix values) [yhgI]	WT	MOPS	Anaerobic	1000	0 / 1	**No**[b]
				MOPS	Aerobic	2250	0 / 1	
suf							Suf	
	Jang & Imlay, 2010	LacZ fusion	Hpx^{-}	Minimal medium	Aerobic	70 mU / mg	0 / 1	y
			$Hpx^{-iscR,oxyR-NI(Suf)}$	Minimal medium	Aerobic	30 mU / mg	0	
	Giel & al, 2006	Microarray (Affymetrix values)	WT	MOPS	Anaerobic	150	0 / 1	**No**[b]
				MOPS	Aerobic	400	0 / 1	
	Mettert & Kiley, 2014	P_{sufA}-*lacZ*(Miller Units)	*iscSUA*$^{-}$	LB	Aerobic	~ 200 Miller Units	0 / 1	y
			furiscSUA^{-}	LB	Aerobic	~ 1200 Miller Units	1	
	Lee & al, 2008	Nuclease S1 (normalized to WT, Default conditions)	WT	LB	Aerobic	1	0 / 1	y
				Dip = 200	Aerobic	11	1	
		Nuclease S1 (normalized to WT, Default conditions)	WT	Dip = 200	Aerobic	11	1	y
			furiscR^{-}	Dip = 200	Aerobic	0.7	0	
		Nuclease S1 (normalized to WT, Default conditions)	WT	LB	Aerobic	1	0 / 1	y
				LB	PMS = 100 µM	58	0 / 1 / 2	

b The experimentally observed changes are under the regulatory threshold affecting IscR activity in our model.

Model predictions

We have generated all mutants induced by single, double, triple . . . KO mutations. The results can be found, alongside the source code at the following location: `https://gitlab.com/Laurent.Tichit/fe-s-cluster-biogenesis-logical-model/`

References

[Andrews et al., 2003] Andrews, S., Robinson, A., and Rodríguez-Quiñones, F. (2003). Bacterial iron homeostasis. *FEMS microbiology reviews*, 27:215–37.

[Angelini et al., 2008] Angelini, S., Gerez, C., Ollagnier-de Choudens, S., Sanakis, Y., Fontecave, M., Barras, F., and Py, B. (2008). NfuA, a new factor required for maturing Fe/S proteins in Escherichia coli under oxidative stress and iron starvation conditions. *The Journal of biological chemistry*, 283(20):14084–91.

[Baez and Shiloach, 2017] Baez, A. and Shiloach, J. (2017). Increasing dissolved-oxygen disrupts iron homeostasis in production cultures of escherichia coli. *Antonie van Leeuwenhoek*, 110(1):115–124.

[Bagg and Neilands, 1987] Bagg, A. and Neilands, J. B. (1987). Ferric uptake regulation protein acts as a repressor, employing iron (II) as a cofactor to bind the operator of an iron transport operon in Escherichia coli. *Biochemistry*, 26(17):5471–7.

[Beauchene et al., 2017] Beauchene, N. A., Mettert, E. L., Moore, L. J., Keleş, S., Willey, E. R., and Kiley, P. J. (2017). O2 availability impacts iron homeostasis in Escherichia coli. *Proceedings of the National Academy of Sciences of the United States of America*, 114(46):12261–12266.

[Beauchene et al., 2015] Beauchene, N. A., Myers, K. S., Chung, D., Park, D. M., Weisnicht, A. M., Keleş, S., and Kiley, P. J. (2015). Impact of Anaerobiosis on Expression of the Iron-Responsive Fur and RyhB Regulons. *mBio*, 6(6):e01947–15.

[Chareyre and Mandin, 2018] Chareyre, S. and Mandin, P. (2018). Bacterial Iron Homeostasis Regulation by sRNAs. In *Regulating with RNA in Bacteria and Archaea*, volume 6, pages 267–281. American Society of Microbiology.

[Desnoyers et al., 2009] Desnoyers, G., Morissette, A., Prévost, K., and Massé, E. (2009). Small RNA-induced differential degradation of the polycistronic mRNA iscRSUA. *EMBO Journal*, 28(11):1551–1561.

[Giel et al., 2006] Giel, J. L., Rodionov, D., Liu, M., Blattner, F. R., and Kiley, P. J. (2006). IscR-dependent gene expression links iron-sulphur cluster assembly to the control of O2-regulated genes in Escherichia coli. *Molecular microbiology*, 60(4):1058–75.

[González-Flecha and Demple, 1997] González-Flecha, B. and Demple, B. (1997). Transcriptional regulation of the Escherichia coli oxyR gene as a function of cell growth. *Journal of bacteriology*, 179(19):6181–6.

[Hartmann and Braun, 1981] Hartmann, A. and Braun, V. (1981). Iron uptake and iron limited growth of Escherichia coli K-12. *Archives of microbiology*, 130(5):353–6.

[Imlay, 2013] Imlay, J. A. (2013). The molecular mechanisms and physiological consequences of oxidative stress: lessons from a model bacterium. *Nature reviews. Microbiology*, 11(7):443–54.

[Jang and Imlay, 2010] Jang, S. and Imlay, J. A. (2010). Hydrogen peroxide inactivates the Escherichia coli Isc iron-sulphur assembly system, and OxyR induces the Suf system to compensate. *Molecular microbiology*, 78(6):1448–67.

[Keyer and Imlay, 1996] Keyer, K. and Imlay, J. A. (1996). Superoxide accelerates DNA damage by elevating free-iron levels. *Proceedings of the National Academy of Sciences of the United States of America*, 93(24):13635–40.

[Klebba et al., 1982] Klebba, P. E., McIntosh, M. A., and Neilands, J. B. (1982). Kinetics of biosynthesis of iron-regulated membrane proteins in Escherichia coli. *Journal of bacteriology*, 149(3):880–888.

[Lee et al., 2008] Lee, K.-C., Yeo, W.-S., and Roe, J.-H. (2008). Oxidant-responsive induction of the suf operon, encoding a Fe-S assembly system, through Fur and IscR in Escherichia coli. *Journal of bacteriology*, 190(24):8244–7.

[Levine and Hwa, 2008] Levine, E. and Hwa, T. (2008). Small RNAs establish gene expression thresholds. *Current Opinion in Microbiology*, 11(6):574–579.

[Li and Imlay, 2018] Li, X. and Imlay, J. A. (2018). Improved measurements of scant hydrogen peroxide enable experiments that define its threshold of toxicity for escherichia coli. *Free Radical Biology and Medicine*, 120:217–227.

[Mandin et al., 2016] Mandin, P., Chareyre, S., and Barras, F. (2016). A Regulatory Circuit Composed of a Transcription Factor, IscR, and a Regulatory RNA, RyhB, Controls Fe-S Cluster Delivery. *mBio*, 7(5):e00966–16.

[Prévost et al., 2007] Prévost, K., Salvail, H., Desnoyers, G., Jacques, J. F., Phaneuf, E., and Massé, E. (2007). The small RNA RyhB activates the translation of shiA mRNA encoding a permease of shikimate, a compound involved in siderophore synthesis. *Molecular Microbiology*, 64(5):1260–1273.

[Salvail et al., 2010] Salvail, H., Lanthier-Bourbonnais, P., Sobota, J. M., Caza, M., Benjamin, J.-A. M., Mendieta, M. E. S., Lépine, F., Dozois, C. M., Imlay, J., and Massé, E. (2010). A small RNA promotes siderophore production through transcriptional and metabolic remodeling. *Proceedings of the National Academy of Sciences of the United States of America*, 107(34):15223–8.

[Schwartz et al., 2001] Schwartz, C. J., Giel, J. L., Patschkowski, T., Luther, C., Ruzicka, F. J., Beinert, H., and Kiley, P. J. (2001). IscR, an Fe-S cluster-containing transcription factor, represses expression of Escherichia coli genes encoding Fe-S cluster assembly proteins. *Proceedings of the National Academy of Sciences of the United States of America*, 98(26):14895–900.

[Seaver and Imlay, 2001a] Seaver, L. C. and Imlay, J. A. (2001a). Alkyl hydroperoxide reductase is the primary scavenger of endogenous hydrogen peroxide in Escherichia coli. *Journal of Bacteriology*, 183(24):7173–7181.

[Seaver and Imlay, 2001b] Seaver, L. C. and Imlay, J. a. (2001b). Hydrogen peroxide fluxes and compartmentalization inside growing Escherichia coli. *Journal of bacteriology*, 183(24):7182–9.

[Seo et al., 2014] Seo, S. W., Kim, D., Latif, H., O'Brien, E. J., Szubin, R., and Palsson, B. O. (2014). Deciphering Fur transcriptional regulatory network highlights its complex role beyond iron metabolism in Escherichia coli. *Nature communications*, 5:4910.

[Vinella et al., 2013] Vinella, D., Loiseau, L., de Choudens, S. O., Fontecave, M., and Barras, F. (2013). In vivo [Fe-S] cluster acquisition by IscR and NsrR, two stress regulators in Escherichia coli. *Molecular Microbiology*, 87(3):493–508.

[Wofford et al., 2018] Wofford, J. D., Bolaji, N., Dziuba, N., Outten, F. W., and Lindahl, P. A. (2018). Evidence that a respiratory shield in Escherichia coli protects a low-molecular-mass Fe II pool from O 2 –dependent oxidation. *Journal of Biological Chemistry*, page jbc.RA118.005233.

[Wright et al., 2013] Wright, P. R., Richter, A. S., Papenfort, K., Mann, M., Vogel, J., Hess, W. R., Backofen, R., and Georg, J. (2013). Comparative genomics boosts target prediction for bacterial small RNAs. *Proceedings of the National Academy of Sciences*, 110(37):E3487–E3496.

[Yeo et al., 2006] Yeo, W.-S., Lee, J.-H., Lee, K.-C., and Roe, J.-H. (2006). IscR acts as an activator in response to oxidative stress for the suf operon encoding Fe-S assembly proteins. *Molecular microbiology*, 61(1):206–18.
